# One-Step Solution Deposition of Tin-Perovskite onto a Self-Assembled Monolayer with a DMSO-Free Solvent System

**DOI:** 10.1021/acsenergylett.3c02098

**Published:** 2023-11-22

**Authors:** Ece Aktas, Isabella Poli, Corinna Ponti, Guixiang Li, Andrea Olivati, Diego Di Girolamo, Fahad Ahmed Alharthi, Meng Li, Emilio Palomares, Annamaria Petrozza, Antonio Abate

**Affiliations:** † Department of Chemical, Materials and Production Engineering, University of Naples Federico II, Piazzale Tecchio 80, 80125 Fuorigrotta, Italy; ‡ Center for Nano Science and Technology @Polimi, Istituto Italiano di Tecnologia, via Rubattino 81, 20134 Milano, Italy; § Helmholtz-Zentrum Berlin für Materialien und Energie GmbH, Hahn-Meitner-Platz 1, 14109 Berlin, Germany; ∥ Physics Department, Politecnico di Milano, Piazza L. da Vinci, 32, 20133 Milano, Italy; ⊥ Department of Chemistry, College of Science, King Saud University, Riyadh 11451, Saudi Arabia; # Key Lab for Special Functional Materials of Ministry of Education National & Local Joint Engineering Research Center for High Efficiency Display and Lighting Technology, School of Materials Science and Engineering, Collaborative Innovation Center of Nano Functional Materials and Applications, Henan University, Kaifeng 475004, China; ∇ Institute of Chemical Research of Catalonia (ICIQ-BIST), Av. Països Catalans 16, Tarragona E-43007, Spain; ¶ ICREA, E-08010 Barcelona, Spain

## Abstract

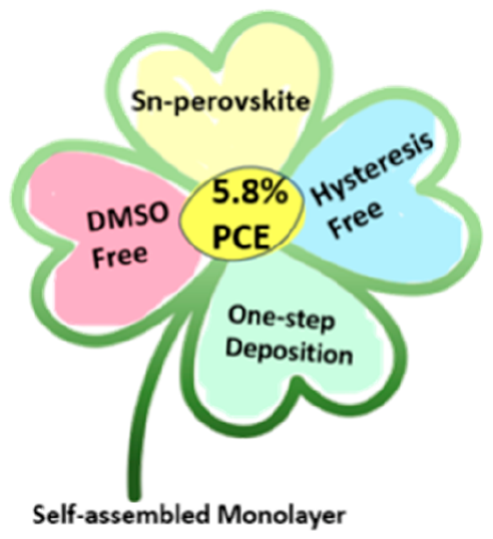

We show for the first time DMSO-free tin-based perovskite solar cells with a self-assembled hole selective contact (MeO-2PACz). Our method provides reproducible and hysteresis-free devices with MeO-2PACz, having the best device PCE of 5.8 % with a *V*_OC_ of 638 mV.

Perovskite solar cells (PSC) have attracted extensive research interest for next-generation solution-processed photovoltaic devices and have taken a big step toward commercialization. Interest in lead-free tin perovskite solar cells (Sn-PSCs) soared once their optoelectronic properties revealed promising alternatives to toxic lead-based PSCs like having low bandgap, large charge-carrier mobility, and low exciton binding energy.^1^ To the best of our knowledge, the highest achieved power conversion efficiency (PCE) of Sn-PSCs is 14.6 % in 2021.^2^ Despite the promising optoelectronic properties and the experience of scientists specialized in the perovskite materials, Sn-PSCs still have not achieved the expected device performance. Hence, some obstacles need to be overcome, such as the undesirable Sn(II) oxidation, the unregulated crystallization rate, and the high hysteresis measured after aging devices.^3,4^

The high-performing Sn-PSCs are generally made in the *pin* sandwich architecture with poly(3,4-ethylenedioxythiophene) (PEDOT:PSS);^2^ however, the hygroscopic and acidic nature of PEDOT:PSS significantly limits the device performance and operational stability under ambient ultraviolet radiation and humidity.^5^ Otherwise, self-assembled molecules (SAMs) recently have been used as hole-selective layers (HSLs) in *pin* structures, thanks to their low-price synthesis pathway^6,7^ and easily functionalized molecular structures,^8^ and demonstrated conformal coverage on large-area substrates.^9^ Additionally, SAM will be a promising HSLs in Sn-PSCs, owing to its ability to modify the contact layers, i.e., indium tin oxide (ITO), and enhance its charge transfer properties. The first application of SAMs as an HSL in Sn-PSCs has been reported by Song et al. and the PCE of the best device reached 6.5 % (the efficiency distribution is 5.2 % ± 0.6 %).^10^ They managed to obtain a uniform dimethyl sulfoxide (DMSO)-processed FASnI_3_-based perovskite film via a two-step sequential deposition method on top of the [2-(3,6-dimethoxy-9*H*-carbazol-9-yl)ethyl]phosphonic acid (MeO-2PACz) SAM. Despite the good performance, a preannealing step at 400 °C for 30 min of the ITO substrates was necessary.^10^

In this study, we demonstrate for the first time that FASnI_3_ perovskite can be successfully deposited on top of the MeO-2PACz SAM with a one-step method using a low-temperature and DMSO-free solvent system of [*N*,*N*-diethylformamide (DEF) and *N*,*N*′-dimethylpropylene urea (DMPU)].^11^ Indeed, we previously showed that DMSO acts as an oxidizing agent for Sn(II) in an acidic medium,^12^ and that it can oxidize Sn(II) species even during the synthesis of the perovskite precursor solution.^13,14^ Besides, DMSO is partially reduced to dimethyl sulfide,^13^ which might influence the crystallization rate of perovskite precursor owing to the low boiling point (37.3 °). We prepared DMSO-free MeO-2PACz SAM-containing Sn-PSCs and compared their performance to control devices that use a water-free PEDOT complex (Figure 1a). The PEDOT-based Sn-PSCs exhibit a champion PCE of 8.7 % with an inverted hysteresis index (HI) of −0.09 while the MeO-2PACz SAM-based device shows a champion PCE of 5.8 % with no hysteresis.

**Figure 1:**
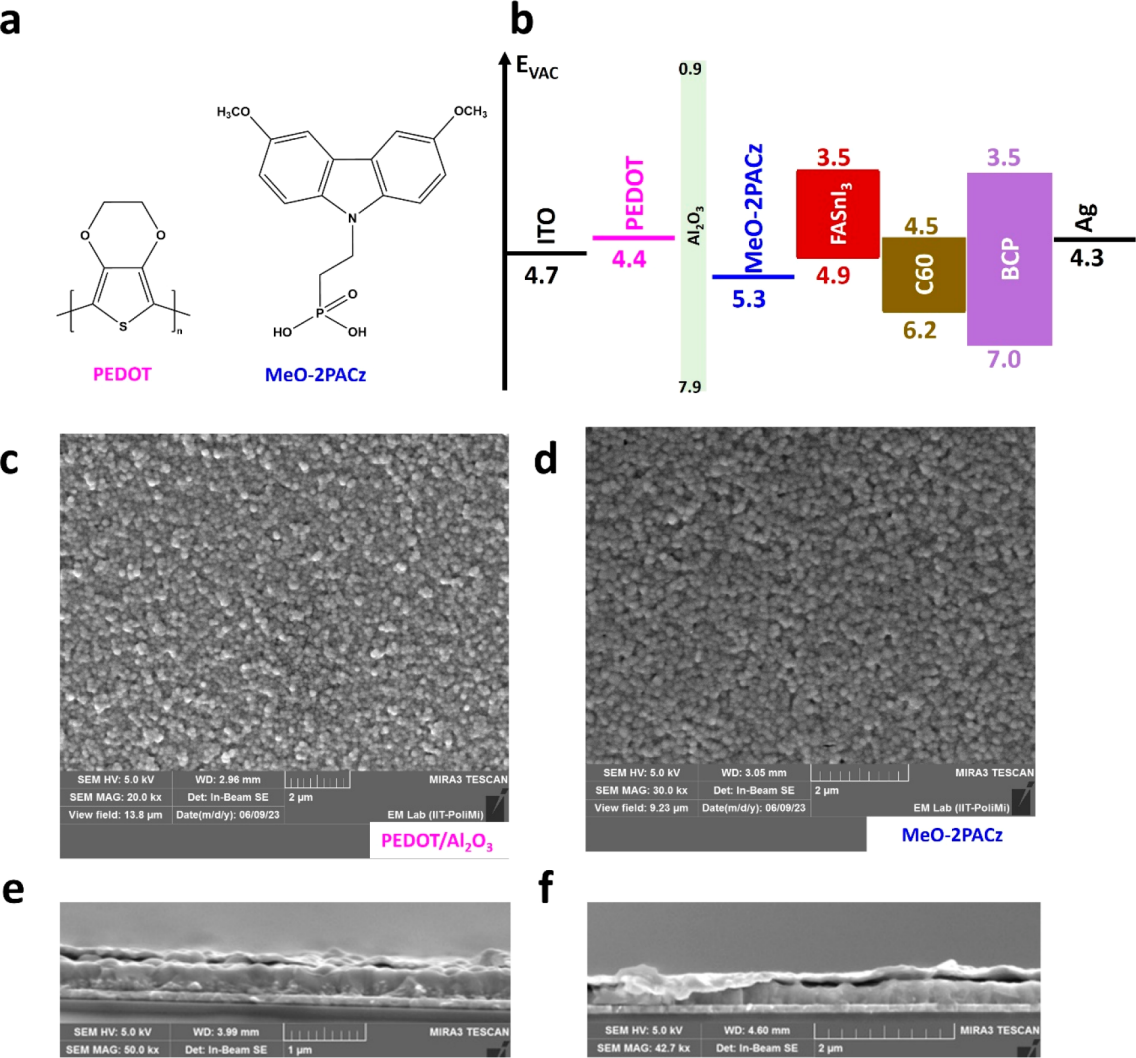
(a) Molecular structures of PEDOT and MeO-2PACz. (b) Energy-band alignment of FASnI_3_ perovskite layer and the various charge transport layers that have been utilized in Sn-PSCs’ devices.^15^ Top-view scanning electron microscopy (SEM) images of the FASI_3_-based perovskite films on (c) PEDOT/Al_2_O_3_ and (d) MeO-2PACz SAM. Cross-sectional images of films on (e) PEDOT/Al_2_O_3_ and (f) MeO-2PACz SAM.

Initially, we spin-coated 0.1 mM of MeO-2PACz SAM solution onto cleaned ITO-covered substrates.^16^ The water-contact angles have been measured to figure out the thickness-dependent wettability of SAM depending on the spin-coater speed (see the Method section for details). The hydrophilicity of MeO-2PACz films slightly improved with increasing spinning speed, with measured contact angles of 53.59 °, 50.77 °, and 47.07 ° for 4000 rpm, 5000 rpm, and 6000 rpm, respectively (Figure S1). In contrast, PEDOT HSL has a more hydrophobic surface (61.80 °)^15^ than MeO-2PACz even after depositing the Al_2_O_3_ interlayer, which is mainly used to improve its wettability. We fabricated ITO/HSL/FASnI_3_/C_60_/BCP/Ag^15^*pin* architecture devices using MeO-2PACz SAM spin-coated at different speeds, finding that the thinner layer (6000 rpm) showed statistically higher efficiency (∼4.5 % average) with better open-circuit voltage (*V*_OC_) and fill factor (FF) (Figure S2). Figure 1b represents the energy band alignment of the materials used in this study. Water-free PEDOT layer has better energy band alignment with the shallow valence band position of FASnI_3_ than MeO-2PACz SAM, which leads to improved hole extraction capabilities and thus increases short-circuit current density (*J*_SC_).^17^

The X-ray diffraction patterns of FASnI_3_ perovskite thin films deposited onto both the PEDOT HSL and the MeO-2PACz SAM exhibit the typical orthorhombic structure, assigned to the crystallographic planes (100), (120), (200), (211), (222), and (300) (Figure S3).^18^ The PEDOT-based perovskite layer exhibits higher crystallinity compared to the MeO-2PACz-based one, as indicated by the sharp increase in the intensity of the 100 and 200 peaks. Higher film crystallinity provides much better optoelectronic properties such as less surface trapping and higher carrier mobility.^19^ As we show in Figure 1c,d, both HSLs provide good coverage with no pinholes, and the crystallites have similar sizes of about 200 nm. Figure 1 panels e and f show the cross-sectional SEM of devices with PEDOT HSL and MeO-2PACz SAM, respectively. Both devices show continuous polycrystalline perovskite layers (∼300 nm). However, the MeO-2PACz SAM-based device shows some delamination at the perovskite/transporting layer interfaces which might limit carrier extraction and device performance.^20^

Determining the accurate PCE of the Sn-PSCs can be notoriously difficult, owing to variations in photovoltaic parameters and hysteresis over time. Although the source of hysteresis is still uncertain in Sn-PSCs, ionic migration, carriers trapping–detrapping at interfaces, and choice of contact material can lead to it.^21^ As shown in Figure 2a,b, a forward prebias followed by a reverse scan statistically (over 30 devices) displays a slightly improved fill factor (FF) and PCE for MeO-2PACz, whereas these parameters are dramatically decreased for PEDOT (their corresponding photovoltaic parameters are listed in Tables S1 and S2). The devices with PEDOT show a broader HI distribution, while SAM devices exhibit a narrower HI distribution (Figure 2c) with no significant changes for *J*_SC_ and *V*_OC_ values measured under forward and reverse scans (Figure S4).

**Figure 2:**
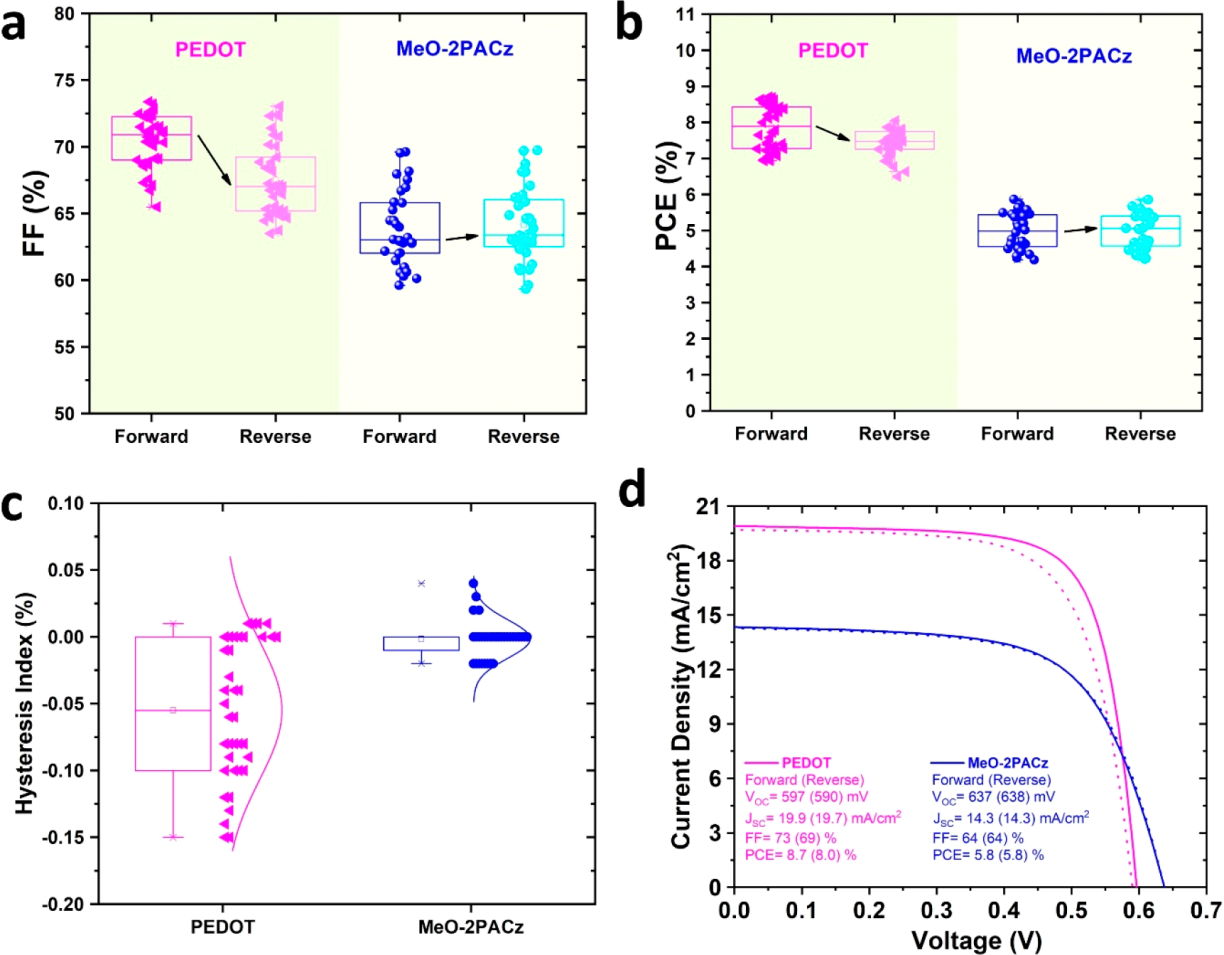
(a) Fill factor, (b) power conversion efficiency, and (c) statistical analysis of the hysteresis index for a device with PEDOT and MeO-2PACz. (d) Best *J*–*V* curves from PEDOT- and MeO-2PACz-based Sn-PSCs.

Figure 2d shows the current density versus voltage (*J–V*) scans and photovoltaic parameters of the best PCEs with PEDOT and MeO-2PACz measured at a scan rate of 100 mVs^–1^ (from forward to reverse bias). The efficiency distribution of PEDOT and MeO-2PACz is 7.8 ± 0.9 % and 5.0 ± 0.8 %, respectively. Notably, the performance of both PEDOT- and MeO-2PACz-based devices increased to record PCEs of 8.7 % and 5.8 %, respectively, after storage in a glovebox environment for 2 weeks (Figure 2d). Integrated short-circuit current density (*J*_SC_) values from incident photon current efficiency are 18.7 and 13.7 mA/cm^2^ for PEDOT and MeO-2PACz, respectively, showing negligible difference with those extracted by the *J–V* curves (Figure S5).

In summary, we demonstrated a simple one-step tin-based perovskite deposition method with a MeO-2PACz SAM by using a DMSO-free solvent system, which led to hysteresis-free solar cells with a champion PCE of 5.8 %. This method has great potential for improving the hysteresis-free SAM-based Sn-PSCs performance with low-temperature processability.

## Supporting Information Available

Device fabrication and characterization methods, including contact angle measurements, XRD, and device performance statistics (PDF)
