# Supplementary material for: One-Step Solution Deposition of Tin-Perovskite onto a Self-Assembled Monolayer with a DMSO-Free Solvent System
Source: ACS Energy Lett. 2023 Nov 22;8(12):5170–4. doi: 10.1021/acsenergylett.3c02098 (PMC10714388; doi:10.1021/acsenergylett.3c02098)
Supplement: Supplementary file 1 — nz3c02098_si_001.pdf [file nz3c02098_si_001.pdf]

Supporting information

for

One-Step Solution Deposition of Tin-Perovskite onto A Self-Assembled Monolayer with A DMSO-Free Solvent System

Authors: Ece Aktas,[1] Isabella Poli,[2] Corinna Ponti,[1] Guixiang Li,[3] Andrea Olivati,[2,4] Diego Di Girolamo,[1] Fahad Ahmed Alharthi,[5] Meng Li,[3,6] Emilio Palomares,[7,8] Annamaria Petrozza,[2] and Antonio Abate [1,3,5*]

Affiliations:

[1]Department of Chemical, Materials and Production Engineering, University of Naples Federico II, Piazzale Tecchio 80, 80125 Fuorigrotta, Italy.

[2]Center for Nano Science and Technology @Polimi, Istituto Italiano di Tecnologia; via Rubattino 81, 20134 Milano, Italy.

[3]Helmholtz-Zentrum Berlin für Materialien und Energie GmbH, Hahn-Meitner-Platz 1, 14109 Berlin, Germany.

[4]Physics Department, Politecnico di Milano, Piazza L. da Vinci, 32, 20133 Milano, Italy

[5]Department of Chemistry, College of Science, King Saud University, Riyadh 11451, Saudi Arabia

[6]Key Lab for Special Functional Materials of Ministry of Education National & Local Joint Engineering Research Center for High Efficiency Display and Lighting Technology, School of Materials Science and Engineering, Collaborative Innovation Center of Nano Functional Materials and Applications, Henan University, Kaifeng 475004, China

[7]Institute of Chemical Research of Catalonia (ICIQ-BIST), Av. Països Catalans 16, Tarragona E-43007, Spain.

[8]ICREA, E-08010 Barcelona, Spain.

[*]Corresponding Author. Email: antonio.abate@unina.it

Materials and Devices

Chemicals:

We purchased water-free PEDOT-complex dispersion in toluene (HTL3) from Clevios™. Formamidinium iodide was purchased from Dyenamo. MeO-2PACz (Product Number: D5798) was purchased from Tokyo Chemical Industry (TCI). Silver shot (1-5 mm, 99.9%) was purchased from Alfa Aesar. C60 was purchased from CreaPhys. All the other chemicals were purchased from Sigma Aldrich and used as received.

Device Fabrication:

The patterned ITO substrates (2.5x2.5cm^2, Kintec, 10 Ohm per square) were washed with in Hellmanex 2% deionised water (DI) solution, DI water, acetone, and ethanol for 15 minutes at 40 °C sequentially. After that, the well-dried ITO substrates were treated in a UV-ozone cleaner for 30 min at 35 °C. Once completing UV-ozone treatment, the cleaned ITO substrates immediately are transferred to N_2 filled glovebox. It is worth mentioning that all of the device fabrication steps were carried out in a highly pure N_2-filled glovebox (glovebox temperature was around 24 °C), and glovebox has never been exposed to Dimethyl sulfoxide (DMSO). Solvent regeneration frequently was applied to avoid solvent vapour. The monolayer of MeO-2PACz is deposited from fresh 1 mM stock solution which was prepared in absolute ethanol. 100 µL of MeO-2PACz solution is statically poured on a pre-cleaned ITO substrates, after 5 sec (give time to it for attaching ITO coated substrate) the solution spun at 6000 rpm for 30 seconds. Then, the MeO-2PACz coated ITO substrates were annealed at 150 °C for 15 min. The freshly prepared diluted PEDOT-complex (1:4 v:v in dry toluene) was deposited/annealed subsequently as in our previous work.[1] The stock solutions of SnI_2 (1.2 M) and $EDAI_2$ (1 M) were prepared in a DMSO-free solvent system from *N*,*N*-diethylformamide:*N*,*N'*-dimethylpropyleneurea (DEF:DMPU, 1:6, v:v) and left overnight in a thermoshaker at 20 °C before preparing the perovskite final solution. The $FASnI_3$ solution (1.2 M in 6:1 DEF:DMPU, with 10% over stoichiometry of SnI_2 and eventually 5% $EDAI_2$) was spun at 500 rpm for 5 seconds and 4000 rpm for 40 seconds. After 21 seconds, 100 µL of diethyl ether (DEE) were dropped on the spinning substrate to induce the perovskite crystallisation. The film was annealed at 100 °C for 30 minutes. As electron selective contact, C_{60} (50 nm) and BCP (7 nm) were subsequently evaporated at a 10^{-6} mbar vacuum level with a deposition rate between 0.1 and 0.3 Å s^{-1}. Following, silver was evaporated through a shadow mask at a rate of 0.1 Å s^{-1} up to 5 nm, increasing to 0.3 Å s^{-1} up to 10 nm and then at 1 Å s^{-1} up to 150 nm. The completed device area is around 0.17 cm^2. The devices always were stored in a glovebox before measuring.

Methods

Contact Angle Measurement: The water contact angle measurements were performed with a Kruss Drop Shape Analysis System DSA25 outside the glovebox. The droplet volume was between 1 and 2 µL.

X-ray Diffraction (XRD) and Scanning Electron Microscope (SEM): Film morphology was characterized using a MIRA3 TESCAN microscope with an accelerating voltage of 5 kV. XRD patterns were recorded with a Bruker D8 Advance diffractometer with Bragg–Brentano geometry equipped with a Cu Ka1(l= 1.544060 Å) anode, operating at 40 kV and 40 mA. All the diffraction patterns were collected at room temperature, with a step size of 0.05 in symmetric scan reflection mode and an acquisition time of 1 s. XRD patterns were recorder on thin films

in an inert environment by means of a Bruker air tight specimen holder with dome like X-ray transparent cap, for environmentally sensitive materials.

Current Density-Voltage Scan (*J-V*): *J-V* curves were measured with a commercial apparatus (Arkeo-Ariadne, Cicci Research s.r.l.) based on a ring of 12 LEDs ranging from 300 to 1000 nm and calibrated to fit class A and a 4-wire source meter.

Incident Photon to Current Efficiency (IPCE): The IPCE spectrum was measured with a commercial apparatus (Arkeo-Ariadne, Cicci Research s.r.l.) based on a 300-Watt xenon lamp, able to acquire a spectrum from 300 to 1000 nm.

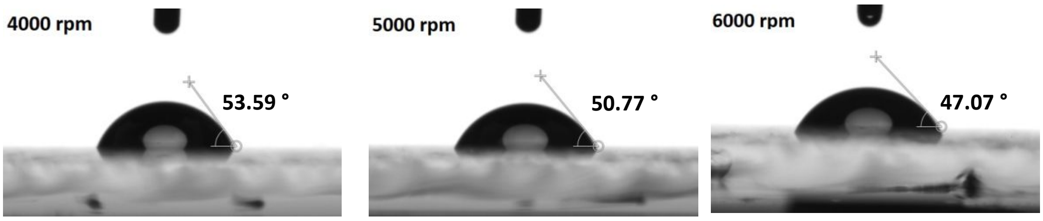

Figure S1. *Water Contact angles on top of ITO/MeO-2PACz films with various deposition speeds.*

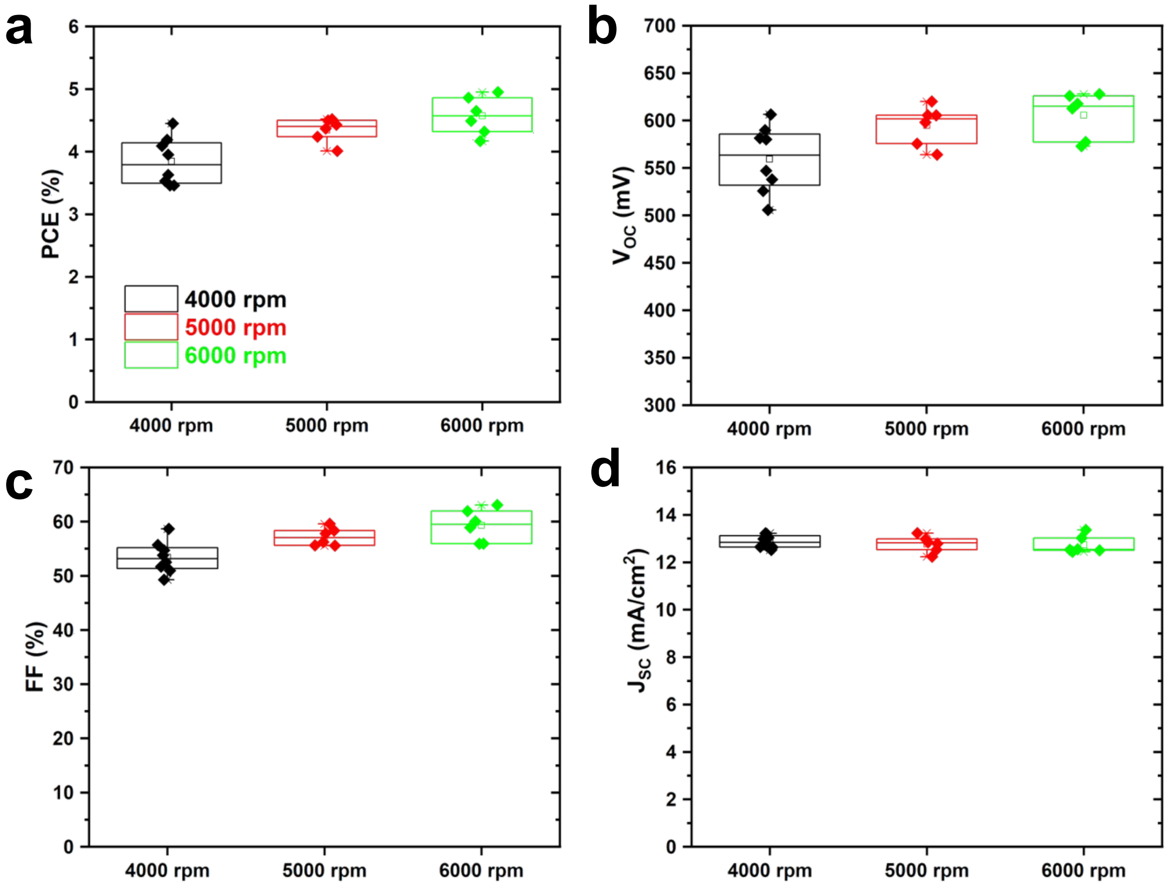

Figure S2. *Device performance statistics of a total 20 devices based on various deposition speeds of MeO-2PACz monolayer from fresh devices.*

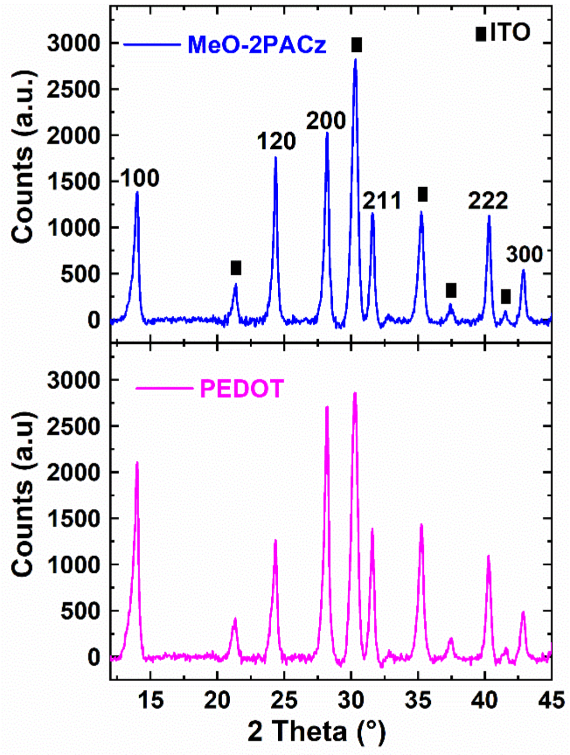

Figure S3. *X-ray diffraction patterns of $FASnI_3$ film with 5% mol ethylenediammonium diiodide ($EDAI_2$) and 10% excess SnI_2 on top of ITO/PEDOT/Al_2O_3 and ITO/MeO-2PACz.*

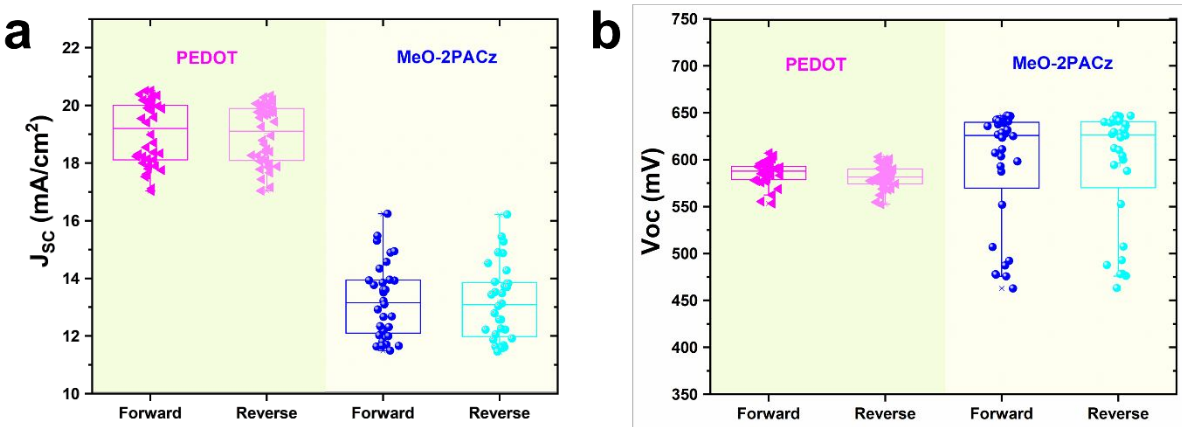

Figure S4. *Forward and reverse scan statistics of device performance from PEDOT and MeO-2PACz based Sn-PSCs after two weeks.*

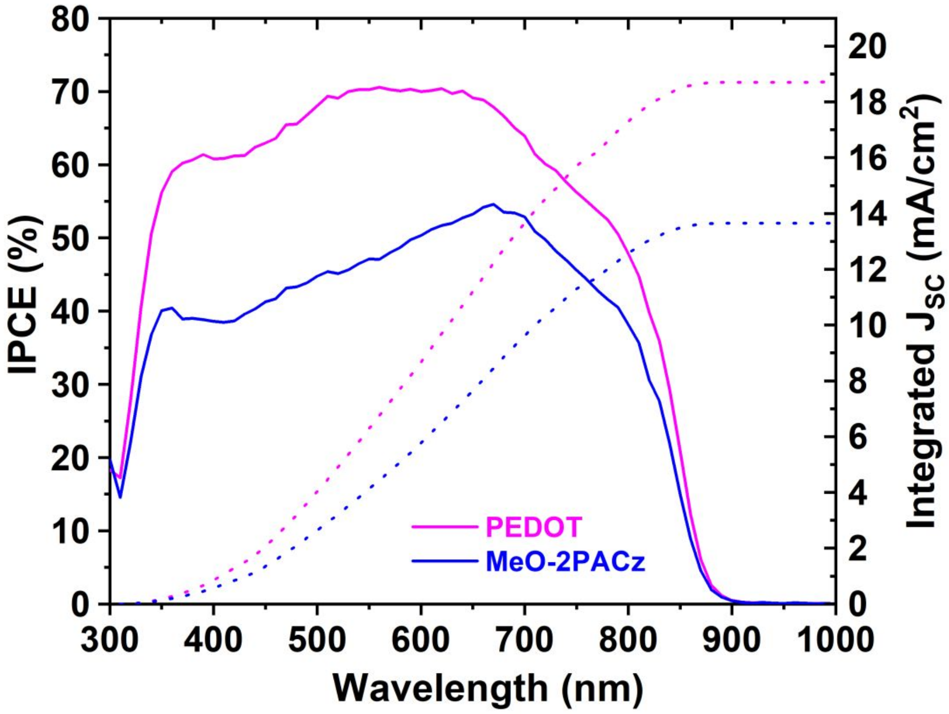

Figure S5. *Corresponding incident photon current efficiency curves which show integrated current density in agreement with values from the J-V curves of PEDOT and MeO-2PACz based Sn-PSCs.*

Table S1. *Photovoltaic parameters of* ***PEDOT****-based Sn-PSCs with forward (FW) and reverse (RV) scan after two weeks.*

Device Number	V_{OC}_FW (mV)	J_{SC}_FW (mA/cm^2)	FF_FW (%)	PCE_FW (%)	V_{OC}_RV (mV)	J_{SC}_RV (mA/cm^2)	FF_RV (%)	PCE_RV (%)	HI (%)
1	607	18.9	67	7.7	601	18.9	65	7.3	-0.05
2	591	20.1	70	8.3	583	19.9	65	7.5	-0.10
3	598	19.4	71	8.2	591	19.2	67	7.6	-0.08
4	597	19.9	73	8.7	590	19.7	69	8.0	-0.09
5	585	17.5	70	7.2	580	17.4	68	6.9	-0.04
6	580	17.9	67	6.9	574	17.9	65	6.7	-0.03
7	576	18.0	67	6.9	568	17.9	64	6.5	-0.06
8	577	18.1	68	7.1	571	18.1	66	6.8	-0.04
9	585	20.2	72	8.5	578	20.0	67	7.7	-0.10
10	590	19.9	71	8.3	578	19.8	65	7.4	-0.12
11	591	19.9	71	8.3	586	19.9	67	7.8	-0.06
12	602	19.6	69	8.1	598	19.6	67	7.8	-0.04
13	585	20.5	72	8.6	569	20.3	65	7.5	-0.15
14	590	20.3	69	8.3	575	20.1	63	7.3	-0.14
15	588	20.5	71	8.6	578	20.3	65	7.6	-0.13
16	593	20.4	71	8.6	582	20.2	66	7.8	-0.10
17	592	19.9	71	8.4	588	19.8	67	7.8	-0.08
18	600	19.8	70	8.3	596	19.7	66	7.7	-0.08
19	596	19.5	69	8.0	590	19.4	65	7.4	-0.08
20	592	19.9	72	8.5	584	19.8	67	7.7	-0.10
21	583	20.3	73	8.6	569	20.1	67	7.7	-0.12

22	578	20.1	73	8.4	562	19.9	65	7.3	-0.15
23	587	20.1	69	8.1	578	20.1	64	7.4	-0.09
24	590	19.8	72	8.4	584	19.7	68	7.8	-0.08
25	585	17.1	73	7.3	584	17.2	73	7.3	0
26	553	18.1	71	7.1	552	18.1	71	7.1	0
27	578	18.2	69	7.3	577	18.3	69	7.3	0
28	555	18.2	69	6.9	555	18.2	68	6.9	0
29	569	18.3	71	7.4	568	18.4	71	7.4	0
30	577	18.3	72	7.6	576	18.4	72	7.7	0.01
31	579	18.5	69	7.4	577	18.6	67	7.3	-0.01
32	562	18.7	69	7.2	562	18.8	69	7.3	0.01
33	604	17.9	65	7.0	603	18.1	65	7.1	0.01
34	601	18.4	71	7.8	599	18.5	70	7.8	0
35	586	17.8	73	7.6	586	17.9	72	7.5	-0.01
36	595	17.6	71	7.4	594	17.7	71	7.5	0.01
37	583	17.7	70	7.2	582	17.8	70	7.2	0
38	591	17.0	72	7.2	590	17.0	72	7.2	0
Average	586±21	19.0±1.5	70±3	7.8±0.9	580±23	18.9±1.4	67±5	7.4±0.6	-0.05

***Table S2**. Photovoltaic parameters of **MeO-2PACz**-based Sn-PSCs with forward (FW) and reverse (RV) scan after two weeks.*

Device Number	V_{OC}_FW (mV)	J_{SC}_FW (mA/cm^2)	FF_FW (%)	PCE_FW (%)	V_{OC}_RV (mV)	J_{SC}_RV (mA/cm^2)	FF_RV (%)	PCE_RV (%)	HI (%)
1	639	12.3	63	4.9	640	12.2	65	5.1	0.04
2	598	11.6	60	4.2	600	11.6	61	4.2	0
3	627	12.0	61	4.6	627	11.9	63	4.7	0.02
4	552	13.5	63	4.7	553	13.5	63	4.7	0
5	492	14.9	63	4.6	493	14.9	63	4.6	0
6	463	14.9	63	4.3	463	14.9	63	4.3	0
7	487	14.6	63	4.5	488	14.5	63	4.5	0
8	507	13.8	64	4.5	507	13.7	65	4.5	0
9	476	16.2	61	4.7	476	16.2	61	4.7	0
10	482	15.3	63	4.6	483	15.3	63	4.6	0
11	477	15.5	64	4.7	478	15.4	65	4.8	0.03
12	611	12.2	61	4.5	612	12.0	61	4.5	0
13	603	11.8	60	4.3	604	11.7	60	4.2	-0.02
14	627	11.7	60	4.4	628	11.6	59	4.3	-0.02
15	625	13.9	63	5.5	626	13.8	63	5.4	-0.02
16	587	13.8	64	5.2	588	13.8	64	5.2	0
17	636	13.9	62	5.5	636	13.7	63	5.5	0
18	641	13.9	61	5.4	641	13.9	61	5.4	0
19	629	13.6	63	5.4	629	13.5	63	5.3	-0.02
20	637	14.3	64	5.8	638	14.3	64	5.8	0
21	639	13.6	66	5.7	639	13.4	66	5.6	-0.02
22	642	12.9	66	5.5	642	12.8	66	5.4	-0.02
23	623	13.2	62	5.1	624	13.1	62	5.1	0
24	646	12.7	68	5.6	646	12.6	68	5.5	-0.02
25	642	13.1	67	5.6	642	13.0	66	5.5	-0.02
26	631	12.3	67	5.2	632	12.2	67	5.2	0
27	647	11.9	70	5.4	647	11.9	70	5.4	0
28	641	12.7	69	5.6	640	12.6	70	5.6	0

29	607	11.6	65	4.6	610	11.6	66	4.7	0.02
30	593	11.7	62	4.3	594	11.6	62	4.3	0
31	647	11.5	67	5.0	647	11.4	68	5.0	0
32	641	12.3	68	5.4	641	12.3	69	5.4	0
Average	594±53	13.2±3	64±6	5.0±0.8	594±53	13.1±3.1	64±6	5.0±0.8	0

References

(1) Di Girolamo, D.; Aktas, E.; Ponti, C.; Pascual, J.; Li, G.; Li, M.; Nasti, G.; Alharthi, F.; Mura, F.; Abate, A. Enabling Water-Free PEDOT as Hole Selective Layer in Lead-Free Tin Perovskite Solar Cells. *Mater. Adv.* **2022**, 9083–9089. https://doi.org/10.1039/d2ma00834c.
